# Grade-lymph node ratio predicts the survival of breast cancer in different molecular types: A surveillance, epidemiology, and end results population-based analysis

**DOI:** 10.1097/MD.0000000000016436

**Published:** 2019-07-12

**Authors:** Chaoqun Liu, Huiyao Li, Ran Zhuo, Lijun Wang, Lihua He, Qiqi Ruan, Xiaoyi Luan, Xiujuan Mo, Yi Sun

**Affiliations:** a Department of Nutrition, School of Medicine, Jinan University, Guangzhou, Guangdong; b Department of Toxicology, Guangzhou Key Laboratory of Environmental Pollution and Health Risk Assessment, School of Public Health, Sun Yat-Sen University, Guangzhou; c Department of Clinical Medicine, International School, Jinan University, Guangzhou, Guangdong; d Department of Environmental Health and Occupational Medicine, School of Public Health, Guilin Medical University, Guilin, Guangxi, China.

**Keywords:** breast cancer, disease-specific survival, metastatic lymph node ratio, SEER

## Abstract

The prognostic value and conflicting results of metastatic lymph node ratio (mLNR) on breast cancer have aroused an increasing concern. We aimed to evaluate the imperative of mLNR classification and prognostic factors in breast cancer with molecular subtypes.

This study uses the database of surveillance, epidemiology, and end results (SEER) to investigate the imperative for reliable mLNR classification and critical prognostic factors in breast cancer with different molecular subtypes.

The prognostic characteristics for disease-specific survival (DSS) of breast cancer were investigated in the SEER cohort (n = 3651). mLNR (*P* = .017) and histology grade (*P* < .001) were independent factors. A novel grade-lymph node ratio (G-R) staging system was proposed for breast cancer prognosis. The receiver operating characteristic curves revealed that the G-R staging system had an accurate 1-, 3-, and 5-year DSS prediction. Further stratification analysis with molecular subtypes of breast cancer (Luminal and TNBC) first proved robust prognostic values of the G-R staging system among molecular subtypes.

The current population-based cohort demonstrated the capacity of mLNR serving as a critical prognostic factor. Also, G-R staging system has the potential to be regarded as reliable classification for breast cancer patients with different molecular subtypes.

## Abstract 2

Supplemental Digital Content is available in the text

## 1 Introduction

Breast cancer is one of the most common primary malignancies and the causes of cancer death. In the United States, 255,180 new cancer cases and 41,070 cancer deaths were found in 2017.^[1]^ The incidence rate of breast cancer was slightly increased from 2004 to 2013 and the death rate for female breast cancer dropped by 38% from 1989 to 2014.^[1,2]^ Age, local tumor size, regional lymph node status, distant metastasis prognostic factors, and molecular types of breast cancer are crucial prognostic indicators for breast cancer and they can be divided into 4 intrinsic molecular subtypes (Luminal A and B, human epidermal growth factor receptor 2 [HER2]-enriched, basal-like, and normal-like).^[3]^

The American Joint Committee on Cancer (AJCC) staging system determined the most important prognostic determinants of breast cancer with the local tumor size, regional lymph node status, and distant metastasis.^[4]^ However, a growing number of studies suggested that lymph node ratios, defined as absolute number of positive lymph nodes (PLNs) divided by the total number of examined lymph nodes (TLNs), may be more accurate for predicting prognostic value because of the adjustment for variability in nodal assessment.^[5–7]^ Till now, no consensus has been reached on the robust and reproducible classification of metastatic lymph node ratio (mLNR),^[8]^ and limited studies revealing the prognostic value of mLNR in breast cancer patients with different molecular subtypes have been reported.^[9,10]^

In order to evaluate the imperative of mLNR classification and prognostic factors in breast cancer with molecular subtypes, we analyzed a population cohort from the surveillance, epidemiology, and end results (SEER) registries in this study.

## 2 Materials and methods

### 2.1 Patients

The information of current retrospective research was collected from the SEER database for patients with breast cancer diagnosed between 1990 and 2013. A total of 3651 patients diagnosed with breast cancer in the SEER database were included who met the following criteria:

(1) year of diagnosis before 2013;
(2) pathologically confirmed that the patients have no history of other malignancies;
(3) patients without distant metastasis;
(4) patients with >1 involved lymph nodes and disease-specific survival (DSS).

This study was approved by the ethics committee of the First Affiliated Hospital of Jinan University.

### 2.2 Statistical analysis

The clinic pathological characteristics of patients were collected as follows: age at diagnosis, gender, race, surgery, tumor site, size, histology, grade, depth of invasion, number of PLN and TLN, AJCC stages, as well as estrogen receptor (ER), progesterone receptor (PR), and HER2 status, classification of mLNR (defined as PLN divided by TLN), and DSS, which was defined as the period from surgery to cancer-related death or the last follow-up, served as the primary endpoints.

X-tile plots (X-tile software version 3.6.1, Yale University School of Medicine, New Haven, CT) were used to categorize the patients with mLNR higher than 0%, and the remaining patients were subsequently divided into 3 categories with the cutoff points of 0.19 and 0.60.^[11]^

Prognostic factors for DSS were identified by log-rank test and the COX proportional hazards regression analysis. Kaplan–Meier method was conducted for DSS estimation and survival curves were validated by the log-rank test. Discrimination of mLNR, grade-lymph node ratio (G-R) staging system and AJCC staging system were displayed with receiver operating characteristic (ROC) curves.

All analyses were carried out by the software statistical package for social sciences (SPSS) version 20.0 (Chicago, IL). Two-sided *P* values of less than .05 were considered to be statistically significant.

## 3 Results

### 3.1 Characteristics of the patients

Three thousand six hundred fifty-one patients between 1990 and 2013 in the SEER database were included in our study cohort and satisfied all the inclusion criteria. As described in Table 1 , female patients comprised most of the population (99.6%). Caucasian patients were in the majority in the study. Grade II (36.5%) and III (37.4%) comprised a dominant proportion in all of the patients, and most patients were diagnosed as the AJCC stage 1 (37.6%). The mean number of PLN was 2.0 ± 4.2, and the mean TLN was 10.5 ± 8.5. Most patients were diagnosed as ER-positive (72.1%), PR-positive (60.0%), or HER-2-unknown (87.7%).

**Table 1:**
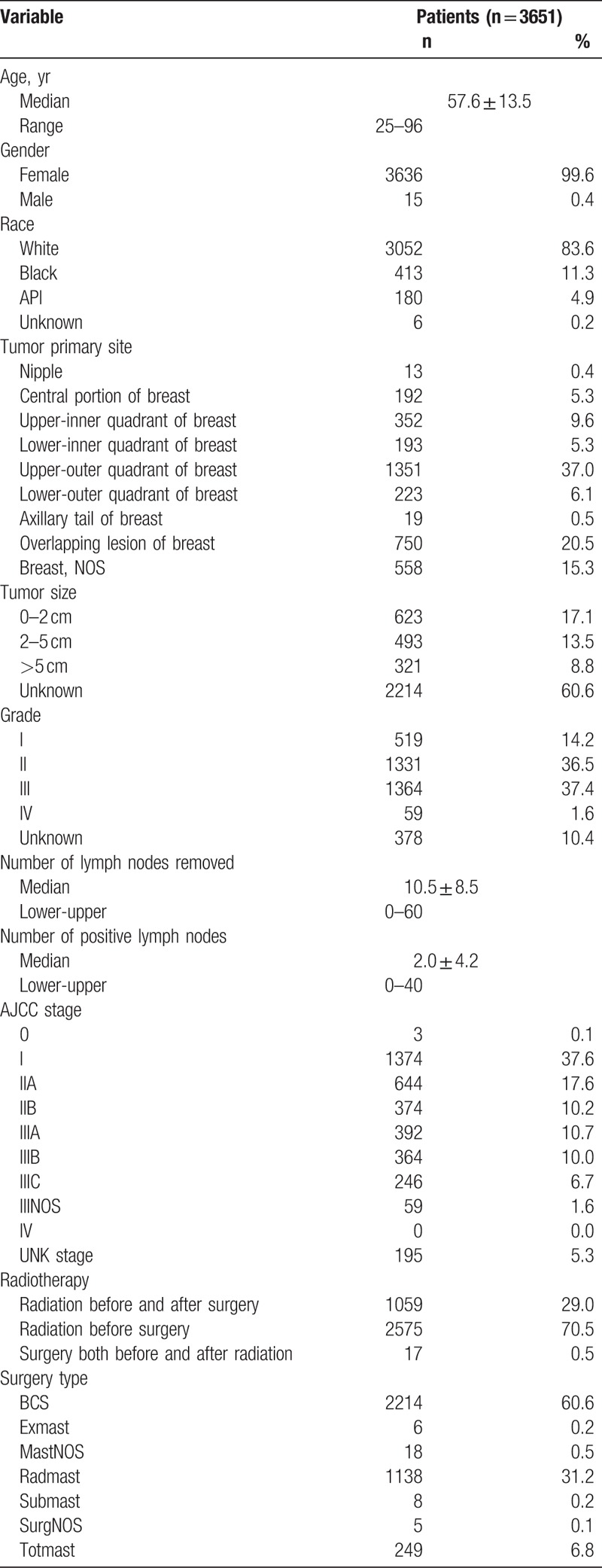
Characteristics of breast cancer patients.

**Table 1 (Continued):**
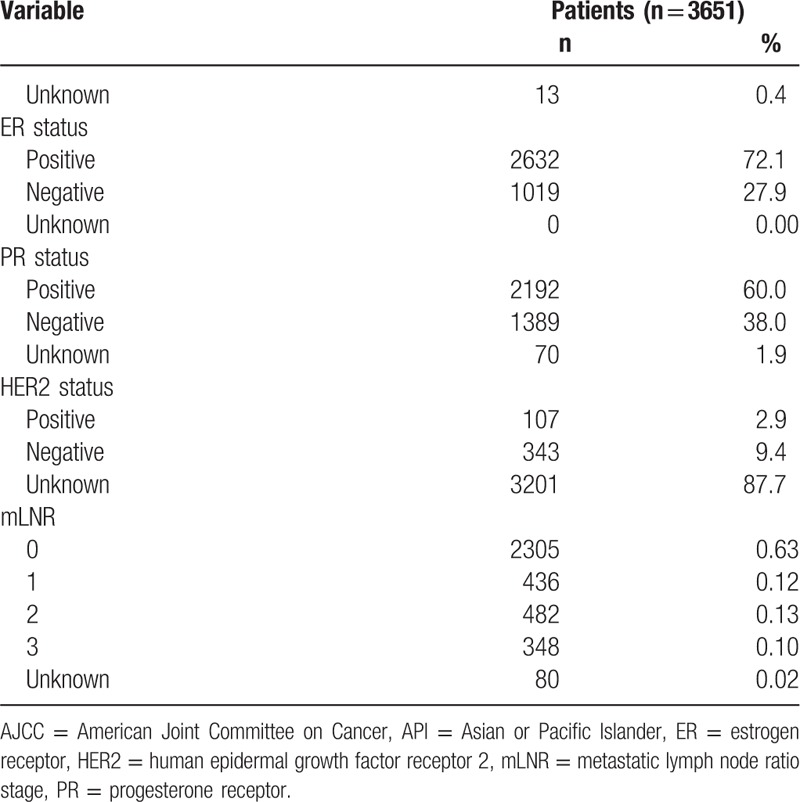
Characteristics of breast cancer patients.

### 3.2 mLNR and survival rates

**Figure 1:**
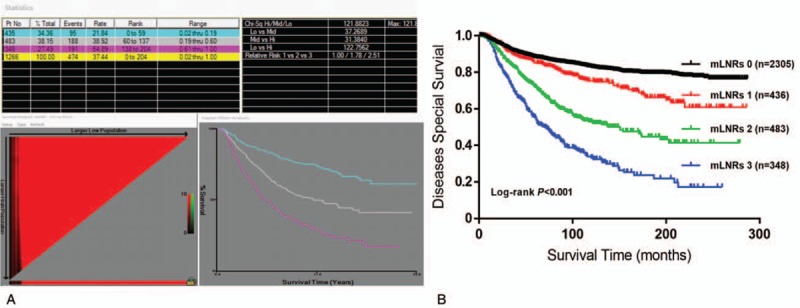
The cutoff points for mLNR identified by X-tile analysis (A), and validated by Kaplan–Meier Curve (B). DSS = disease-specific survival, mLNRs = metastatic lymph node ratio stage.

The mean DSS for all patients was 214 months, and the 1-year, 3-year, and 5-year DSS was 98%, 88%, and 82%, respectively. mLNR was defined as the ratio of the metastatic lymph node number divided by the number of total lymph nodes examined. The best cutoff points of mLNR indicated by X-file were 0.19 and 0.60 (Fig. 1A). We subsequently divided the study cohort into 4 groups, mLNR 0: mLNR = 0%; mLNR 1: 0 < mLNR < 19%; mLNR 2: 19% < mLNR < 60%; mLNR 3: mLNR > 60%. The 5-year survival of DSS were 88%, 85%, 72%, and 55% for mLNR 0, 1, 2, and 3, respectively (*P* < .001, Fig. 1B).

### 3.3 Prognostic factors of breast cancer patients

**Figure 2:**
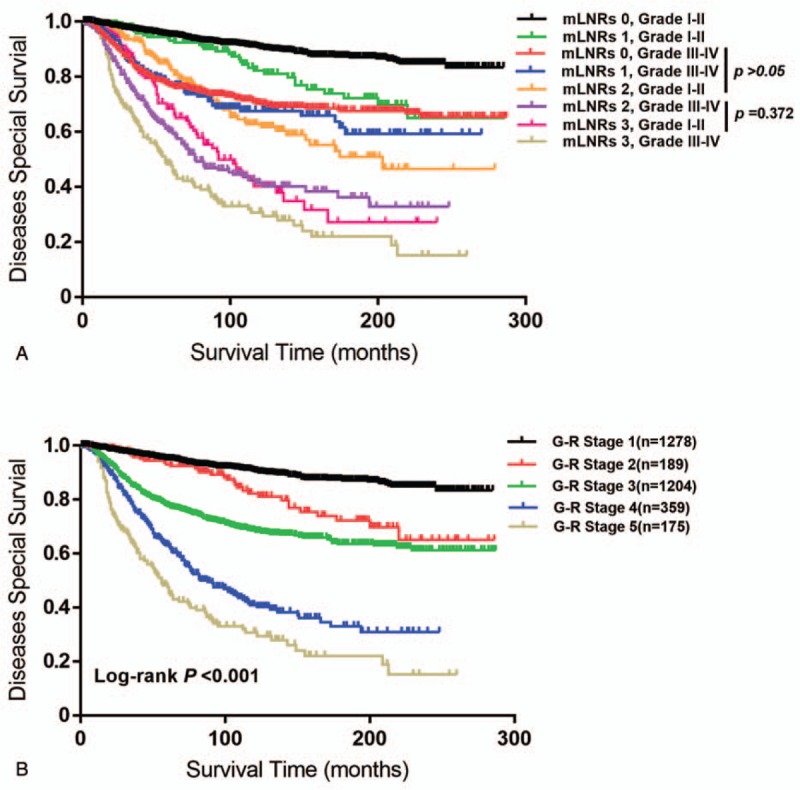
The breast cancer patients were classified into 8 groups and validated by Kaplan–Meier Curve (A). The study cohort population was divided into 5 groups and the classification was validated by Kaplan–Meier Curve (B). DSS = disease-specific survival, G-R stage = grade-lymph node ratio, mLNR = metastatic lymph node ratio stage.

The results of prognostic factors for DSS using log-rank test and the COX regression analysis were displayed in Table 2. In univariate analysis, age at diagnosis, race, tumor size, grade, ER/PR/HER-2 status, and mLNR were risk factors for DSS. The significant factors above were involved in multivariate analysis, and the results figured out the Grade (*P* < .001) and mLNR (*P* = .017) were still independent and significant risk factors for DSS (Table 2, Supplementary Table 1, which illustrate the effect of Grade and mLNR classification on DSS). Based on the Grade and mLNR statuses, the breast cancer patients were divided into 8 groups, Group 1: mLNR 0 and Grade I-II; Group 2: mLNR 0 and Grade III-IV; Group 3: mLNR 1 and Grade I-II; Group 4: mLNR 1 and Grade III-IV; Group 5: mLNR 2 and Grade I-II; Group 6:mLNR 2 and Grade III-IV; Group 7: mLNR 3 and Grade I-II; Group 8: mLNR 3 and Grade III-IV (Fig. 2A). As there was no difference between Group 3, 4, and 5 (*P* > .05 for each of 2 groups) or Group 6 and 7 (*P* = .372), an integrated Grade-mLNR (G-R) staging system for breast cancer patients was proposed at 5 levels: G-R Stage 1, mLNR 0 and Grade I-II; G-R Stage 2: mLNR 0 and Grade III-IV; G-R Stage 3: mLNR 1 and Grade I- IV or mLNR 2 and Grade I-II; G-R Stage 4: mLNR 2 and Grade III-IV or mLNR 3 and Grade I-II; G-R Stage 5: mLNR 3 and Grade III-IV. The Kaplan–Meier analysis showed the 5 G-R Stage levels were significantly different (Fig. 2B).

**Table 2:**
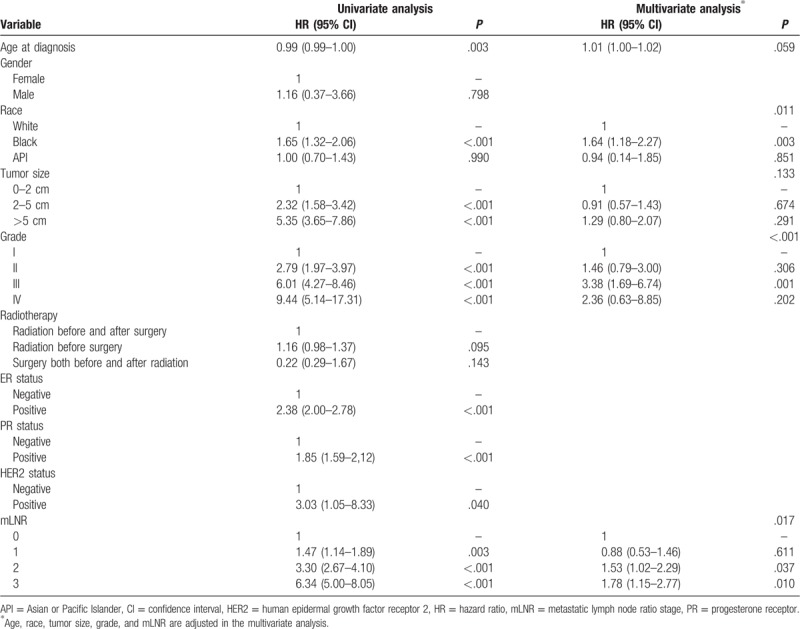
Prognostic characteristics of breast cancer patients for DSS.

### 3.4 Comparison of predictive accuracy for mLNR, G-R staging system, and AJCC staging system

**Figure 3:**
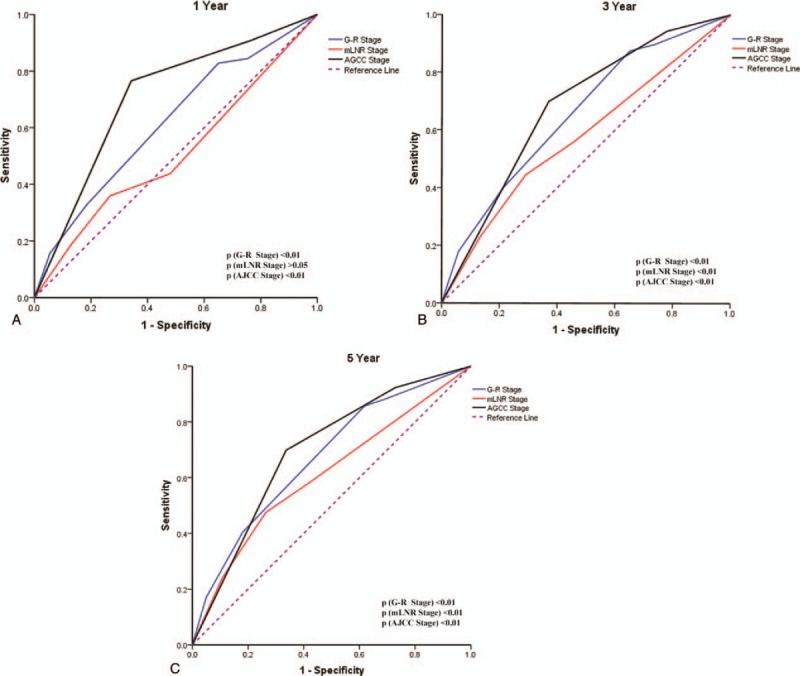
Comparison of the AUC among the G-R staging system, mLNR, and the AJCC staging system to predict DSS at 1-year (A), 3-year (B), 5-year (C). AJCC = American Joint Committee on Cancer, DSS = disease-specific survival, G-R stage = grade-lymph node ratio, mLNR = metastatic lymph node ratio stage.

The predictive accuracy of DSS among mLNR, G-R staging system, and AJCC staging system were valued by the area under the ROC (AUC) curve. As shown in Figure 3 and Table 3, the AUC of AJCC staging system was higher than that of mLNR and G-R staging system. The G-R staging system could also predict prognostic values of 1-, 3-, and 5-year of DSS with statistical significance (all *P* < .001). The above results indicated that G-R staging system could be a good prognostic indicator for breast cancer.

**Table 3:**
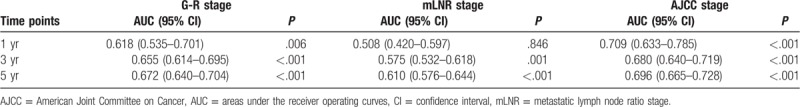
Comparison of predictive accuracy of DSS for G-R staging system, single independent factor and the 7th AJCC staging system in each time point.

### 3.5 Stratification analysis with molecular subtype

**Figure 4:**
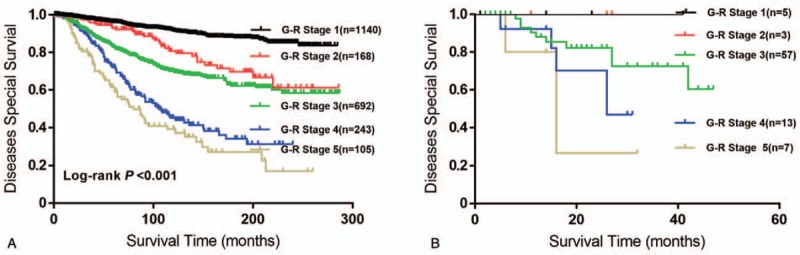
Stratification analysis with molecular subtype of Luminal A/B (A) and TNBC (B) and the classification was validated by Kaplan–Meier Curve. G-R stage = grade-lymph node ratio.

As mentioned above, molecular typing was a prognostic factor for DSS (Table 2). The molecular type distribution of ER and PR varied significantly among different G-R stage (all *P* < 0.001, see Supplementary Table 2, that demonstrates the distribution of receptor expression among patients with different G-R stage). To figure out the effects of molecular subtypes on DSS and G-R staging system, patients were stratified based on their ER, PR, and HER-2 statuses. From this study cohort, there were 2348 Luminal A/B (ER or PR-positive) cases, 41 HER-2 (ER and PR-negative and HER-2-positive) cases, and 85 triple-negative breast cancer TNBC (ER, PR, and HER-2- negative) cases. As with the results of the whole study population, both Luminal A/B (Fig. 4A) and TNBC (Fig. 4B) patients had a worse outcome as the G-R Stage elevated. Data of HER-2 patients were not presented because of the limited case number. These results demonstrated the robust prognostic value of the G-R staging system among molecular subtypes. Besides, race and age were considered as a risk factor in the univariate analysis. We also demonstrated the use of G-R staging system as a predictor of survival with different races and age (see Supplementary Figs. 1 and 2, that illustrates the stratification analysis with race and age).

## 4 Discussion

In this study, we proposed a novel G-R staging system to evaluate the survival in breast cancer with different molecular typing. Based on a large cohort study in SEER dataset, we identified several independent risk factors for prognostic status including grading and mLNR after adjusting other prognostic factors. A robust and reproducible classification of mLNR was validated and a new G-R staging system was proposed and proven to have precise DSS prediction in all study cohorts and especially in each molecular typing patient.

An increasing number of studies were focused on the prognostic value of mLNR in multiple types of cancer especially in breast cancer.^[5,12]^ The current AJCC staging system for breast cancer relies on the absolute number of malignant lymph nodes, but the heterogeneity results from the lymph node examinations may cause variations between centers because of the differences in procedures of lymph node clearance, physical and specimen examination findings.^[9]^ Compared with the lymph node number, many studies have indicated that mLNR is a more accurate prognostic factor of survival in breast cancer.^[13,14]^ The mLNR system was developed and evaluated largely as a way to mitigate low lymph node counts^[15,16]^ and provides a standardized value for comparison of prognosis across patients, regardless of whether very few or very many lymph nodes were retrieved. The mLNR classification is an easily available and inexpensive prognostic factor and therefore has the potential for wide clinical application.^[17]^ As a result of its heralded prognostic value, some have even suggested that mLNR should be considered for incorporation into the AJCC staging system for breast cancer.^[18–20]^ Determining which cutoff value will be the most reliable for predicting breast cancer patients’ needs is critical. This requires a large cohort study to stratify and evaluate different mLNRs on the breast cancer prognosis and find out the small differences in prognostic outcomes. Then the most reliable cutoff points could be produced based on their similar prognostic effect, but the designation of cutoff points value has varied among studies for a staging classification.^[6,15,16,21,22]^ A previous study on universal cutoff points of mLNR in breast cancer was 0.20 and 0.65 for low/high-risk group.^[6]^ Our study proposed similar cutoff values of 0.19 and 0.60 based on X-file plots which were free from predefined assumptions or distributional property. The high- or low-mLNR categories were independent risk factors for DSS in univariate and multivariate analysis showed a significant separation for survival (Table 2 and Fig. 1B).

In the SEER dataset, grade and mLNR were identified as risk factors (Table 2 and Supplementary Table 1, which illustrate the effect of Grade and mLNR classification on DSS). A G-R staging system was proposed to evaluate the survival of breast cancer and the fifth G-R Stage subtype had the worst prognosis among 5 groups (Fig. 2A and B). Furthermore, we compared the predictability of mLNR, G-R staging system, and AJCC staging system by AOC curves. All of the 3 models had the ability of survival prediction for breast cancer (Table 3). However, mLNR model could not predict the 1-year DSS which was probably because it was comprised of single independent factor and the short period of time. G-R staging system was proven to be a better predictor of DSS compared with mLNR model. Although the conventional AJCC staging system presents an optimal tool, G-R staging system could also be a good prognostic indicator for breast cancer. What's more, the prognostic role of G-R staging system has been shown to be effective for gastric cancer patients after neoadjuvant radiotherapy from the SEER database.^[23]^

Commonly used clinical markers of primary breast cancers such as ER status, PR status, and HER2,^[24–26]^ are used to help making decisions about therapy in the metastatic setting. Together, these 3 markers are used to define 4 tumor subtypes: Luminal A and B, HER2-enriched, basal-like, and normal-like. These molecular types of breast cancer have been considered as critical factors for clinical and prognostic phenotype.^[27,28]^ Our findings also revealed the crucial role for DSS (Table 2), and that receptor's expressions in breast cancer patients vary in different G-R stages. A recent study had proposed the prognostic significance of the mLNR with TNBC patients.^[10]^ To the best of our knowledge, our work is the largest study demonstrating the prognosis prediction of mLNR and grading in breast cancer patients with different molecular subtypes. Moreover, we found that the G-R staging system could identify the prognosis of breast cancer patients in Luminal and TNBC subtypes (Fig. 4A and B).

There were still several limitations in our study. First, the lack of critical variables like adjuvant chemotherapy and recurrence types in SEER database restricted our adjustment of confounding factors. Second, HER-2 status could not be found in SEER before 2010 so we lack sufficient data to identify the prognostic value of the G-R Stage in HER-2 subtype. Third, although AJCC staging system has higher AOC score than G-R staging system, a modified staging system based on mLNR, grade, depth of invasion and metastatic status are needed for a more accurate DSS prediction.^[23]^

## 5 Conclusion

In conclusion, our results demonstrated that mLNR is an independent prognostic factor in breast cancer patients and the G-R staging system could be an indication model for DSS among patients with different molecular subtypes. This may throw light upon the optimization for the current TNM classification system. Further prospective multicenter studies are needed to verify G-R staging system in predicting the survival of breast cancer in different molecular subtypes.

## Acknowledgment

The authors thank the efforts of the National Cancer Institute.

## Author contributions

**Conceptualization:** Huiyao Li, Chaoqun Liu, Yi Sun.

**Formal analysis:** Huiyao Li.

**Methodology:** Huiyao Li.

**Software:** Huiyao Li.

**Data curation:** Chaoqun Liu, Yi Sun.

**Writing – Original Draft:** Huiyao Li, Yi Sun.

**Writing – review and editing:** Chaoqun Liu, Ran Zhuo, Lijun Wang, Qiqi Ruan, Lihua He, Xiaoyi Luan, Xiujuan Mo, Yi Sun.

## Supplementary Material

Supplemental Digital Content
