# Supplementary material for: Grade-lymph node ratio predicts the survival of breast cancer in different molecular types: A surveillance, epidemiology, and end results population-based analysis
Source: Medicine (Baltimore). 2019 Jul 12;98(28):e16436. doi: 10.1097/MD.0000000000016436 (PMC6641679; doi:10.1097/MD.0000000000016436)

**Grade-lymph node ratio predicts the survival of breast cancer in different molecular types: a SEER population-based analysis**

Chaoqun Liu, PhD^a^,*, Huiyao Li, PhD ^b^,*

| **Supplementary Table 1: Effect of Grade and mLNR classification on DSS among breast cancer patients** | | |
| --- | --- | --- |
| **Variable** | **Multivariate analysis** | |
|  | HR (95% CI) | *P* |
| Grade |  | **< 0.001** |
| I | 1 | - |
| II | 1.98 (1.41-2.78) | **< 0.001** |
| III | 4.25 (3.06-5.90) | **0.001** |
| Ⅳ | 5.63 (3.41-9.31) | **< 0.001** |
| mLNR |  | **< 0.001** |
| 0 | 1 | - |
| 1 | 1.35 (1.06-1.71) | **< 0.001** |
| 2 | 2.86 (2.38-3.45) | **< 0.001** |
| 3 | 4.31 (3.56-5.22) | **< 0.001** |

| **Supplementary Table 2: Distribution of receptor expression in breast cancer patients with different G-R stage** | | | | | | | | |
| --- | --- | --- | --- | --- | --- | --- | --- | --- |
| **G-R Stage** | **ER** | **n (%)** |  | **PR** | **n (%)** |  | **Her 2** | **n (%)** |
|  | + | - |  | + | - |  | + | - |
| 1 | 1145 (89.6) | 133 (10.4) |  | 976 (77.8) | 279 (22.2) |  | 18 (18.4) | 80 (81.6) |
| 2 | 167 (88.4) | 22 (11.6) |  | 140 (75.7) | 45 (24.3) |  | 6 (20.7) | 23 (79.3) |
| 3 | 667 (55.4) | 537 (44.6) |  | 539 (45.5) | 645 (54.5) |  | 48 (25.8) | 138 (74.2) |
| 4 | 235 (65.5) | 124 (34.5) |  | 189 (53.4) | 165 (46.6) |  | 18 (28.6) | 45 (71.4) |
| 5 | 99 (56.6) | 76 (43.4) |  | 81 (47.6) | 89 (52.4) |  | 4 (20.0) | 16 (80.0) |
| ***P*** | **< 0.001** | |  | **< 0.001** | |  | 0.538 | |


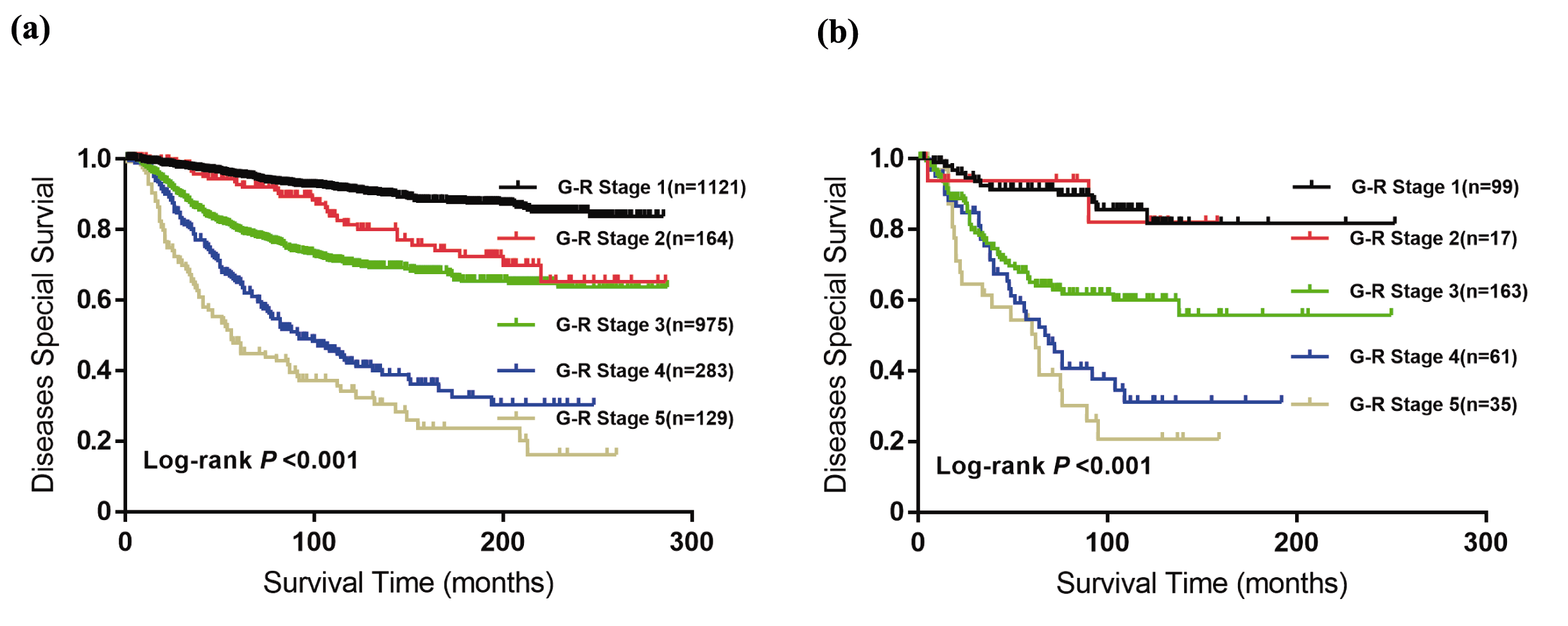


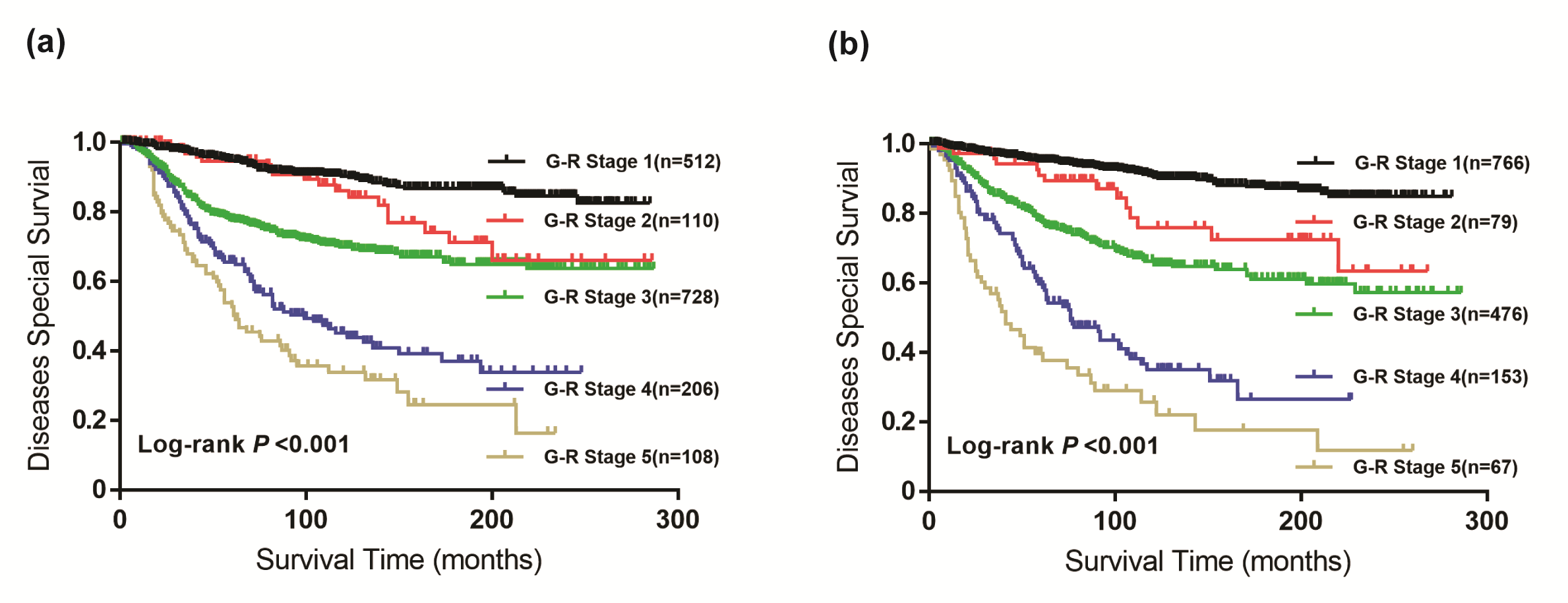

Supplement: Supplemental Digital Content [file medi-98-e16436-s001.doc]
